# Morphological Characters Are Compatible with Mitogenomic Data in Resolving the Phylogeny of Nymphalid Butterflies (Lepidoptera: Papilionoidea: Nymphalidae)

**DOI:** 10.1371/journal.pone.0124349

**Published:** 2015-04-10

**Authors:** Qing-Hui Shi, Xiao-Yan Sun, Yun-Liang Wang, Jia-Sheng Hao, Qun Yang

**Affiliations:** 1 College of life sciences, Anhui Normal University, Wuhu, China; 2 State Key Laboratory of Palaeobiology and Stratigraphy, Nanjing Institute of Palaeontology and Stratigraphy, Chinese Academy of Sciences, Nanjing, China; 3 Sanming College, Sanming, China; Nanjing Agricultural University, CHINA

## Abstract

Nymphalidae is the largest family of butterflies with their phylogenetic relationships not adequately approached to date. The mitochondrial genomes (mitogenomes) of 11 new nymphalid species were reported and a comparative mitogenomic analysis was conducted together with other 22 available nymphalid mitogenomes. A phylogenetic analysis of the 33 species from all 13 currently recognized nymphalid subfamilies was done based on the mitogenomic data set with three Lycaenidae species as the outgroups. The mitogenome comparison showed that the eleven new mitogenomes were similar with those of other butterflies in gene content and order. The reconstructed phylogenetic trees reveal that the nymphalids are made up of five major clades (the nymphaline, heliconiine, satyrine, danaine and libytheine clades), with sister relationship between subfamilies Cyrestinae and Biblidinae, and most likely between subfamilies Morphinae and Satyrinae. This whole mitogenome-based phylogeny is generally congruent with those of former studies based on nuclear-gene and mitogenomic analyses, but differs considerably from the result of morphological cladistic analysis, such as the basal position of Libytheinae in morpho-phylogeny is not confirmed in molecular studies. However, we found that the mitogenomic phylogeny established herein is compatible with selected morphological characters (including developmental and adult morpho-characters).

## Introduction

The family Nymphalidae (Lepidoptera: Papilionoidea) is the largest group of butterflies with about 7,200 species distributed on all continents except Antarctica [1–3]. They are usually medium to large sized butterflies with striking colors, specialized insect-plant interaction and a deep rooted evolutionary history probably 90 million years ago [4, 5]. Owing to their species richness and ecological diversification, nymphalids have been used as model taxa for a wide range of evolutionary and ecological studies [6]. However, the phylogeny of Nymphalidae is one of the most controversial issues in insect studies [7–10]. For instance, in Chou’s taxonomic system, widely adopted in China, the nymphalid butterflies are split into 10 morphological subfamilies [4], while the most commonly adopted classification of the family, proposed by Ackery et al. [11], Harvey [12] and Müller [13], consists of 13 subfamilies. Recently, although efforts have been focused on the phylogeny of Nymphalidae by using morphological, molecular (mitochondrial and nuclear genes), or combined data, problems still exist, particularly about the phylogenetic status of Biblidinae, Pseudergolinae, Cyrestinae, Morphinae, Libytheinae and Charaxinae [14–21]. Common to previous molecular phylogenetic studies are small number of molecular markers used with conflicting results and often weak supports about their phylogenetic relationships.

The insect mitochondrial genomes (mitogenomes) provide abundant molecular markers for phylogenetic and population genetic studies at various hierarchical levels, due to their unique features, such as maternal inheritance, lack of extensive recombination and moderate nucleotide substitution rates [22, 23].

Up to now, existing information about butterfly mtDNAs, especially the complete genomes of mtDNA are still limited. Only 22 nymphalid mitogenomes so far deposited in GenBank (up to Jun 15, 2014) represent eight of the thirteen nymphalid subfamilies. However, the corresponding sequences of the other major nymphalid groups (e.g., Charaxinae, Pseudergolinae, Cyrestinae, Biblidinae and Morphinae) are still lacking. In this study, we sequenced nine complete and two nearly complete mitogenomes of nymphalids, representing nine subfamilies (*Hypolimnas bolina* for Nymphalinae, *Cethosia bibles* for Heliconiinae, *Polyura nepenthes* for Charaxinae, *Ariadne ariadne* for Biblidinae, *Elymnias hypermnestra*, *Lethe dura* and *Callerebia suroia* for Satyrinae, *Parantica aglea* for Danainae, *Cyrestis thyodamas* for Cyrestinae, *Dichorragia nesimachus* for Pseudergolinae and *Stichophthalma howqua* for Morphinae) and compared them with other available nymphalid mitogenomes. A phylogenetic analysis based on mitogenomic data set, assisted with morphological comparison, is attempted to further resolve the nymphalid phylogeny.

## Materials and Methods

### Ethics Statement

All samples of butterfly species used in this study were collected from the mountains or peri-urban areas of China, where no specific permissions were required for these locations or activities. There are no endangered or protected species and all the all samples are common nymphalid butterfly species which are not included in the “List of Protected Animals in China” and other related lists of animal species protection in the world. Thus the sampling in this study did not violate any law, rule or regulation in China and all around the world, requiring no ethical or institutional approval.

### Samples and DNA extraction

The samples of the eleven newly sequenced nymphalid species were collected in China from 2006 to 2012 (S1 Table). After sample collection, fresh individuals were preserved in 100% ethanol and stored at -20°C until used for genomic DNA extraction. Total genomic DNA was extracted from the thorax muscle of a single individual using the Sangon Animal genome DNA Extraction Kit in accordance with the manufacturer instructions (Shanghai, China).

### Primer design, PCR amplification, and sequencing

Insect universal primers were initially used for the amplification of partial fragments of four genes (*cox1*, *cox3*, *cob* and *rrnS* gene) [24–26], approximately 500~700 bp in length. Primers for amplification of the *nad2*, *nad5* and overlapping long fragments were designed via the sequence alignment of all the other sequenced butterfly mitogenomes available, using ClustalX 2.1 and Primer Premier 5.0 software [27, 28]. All primers were synthesized by the Sangon Biotechnology Co. Ltd., Shanghai, China. Six long fragments (*cox1*—*cox3*, *cox3*—*nad5*, *nad5*—*cob*, *cob*—*rrnS*, *rrnS*—*nad2*, *nad2*—*cox1*) were amplified via long PCR technique with the cycling parameters: 30 cycles of denaturation at 95°C for 50 seconds, annealing at 49–55°C (depending on primer pairs) for 50 seconds, and elongation at 68°C for 150 seconds, and a final extension at 68°C for 10 minutes. The PCR products were visualized by electrophoresis on 1.2% agarose gel, then purified using a 3S Spin PCR Product Purification Kit and sequenced directly for the majority of fragments or by cloning the *rrnS—nad2* for some species, with an ABI-377 automatic DNA sequencer. All of the long PCR fragments were sequenced using the primer walking strategy.

### Sequence assembly, gene annotation, and analysis

Sequences from overlapping fragments were initially assembled via the alignment of neighboring fragments using the BioEdit 7.0 [29] and ClustalX 2.1 softwares [27]. Protein-coding genes (PCGs) and ribosomal RNA genes (rRNAs) were identified using ClustalX 2.1 software [27] and the NCBI Internet BLAST search function. Identification of transfer RNA genes (tRNAs) was conducted using software tRNAscan-SE 1.21 (http://lowelab.ucsc.edu/tRNAscan-SE/) [30]. Putative tRNAs that could not be found by tRNAscan-SE were identified by alignment with those of the other butterfly species available. The tandem repeats in the A+T-rich region were predicted using the Tandem Repeats Finder online (http://tandem.bu.edu/trf/trf.html) [31]. Nucleotide composition and codon usage were calculated by MEGA 5.0 software [32]. All three codon positions of the 13 PCGs concatenated nucleotide dataset were tested independently for substitution saturation, by plotting the number of observed substitutions against the genetic distance estimates using MEGA 5.0 software [32]. The rRNA secondary structures were predicted after the models for *Drosophila* [33], *Apis mellifera* [34] and *Manduca sexta* [35]. Stem-loops were named using both the conventions of Gillespie et al. [34] and Niehuis et al. [36, 37] with the former notation first for each time they are mentioned.

### Phylogenetic analysis

Thirty three nymphalid species with complete or nearly complete mitogenome sequence data, including eleven newly determined in this study, were used in the phylogenetic analyses, representing all currently recognized subfamilies of Nymphalidae (Table 1). Three species, *Coreana raphaelis*, *Spindasis takanonis* and *Protantigius superans* from Lycaenidae, being sister group of Nymphalidae, were selected for outgroup comparisons.

**Table 1:** List of the nymphalid species analyzed in this study.

Subfamily	Tribe	Species	Size (bp)	Genbank accession no.	Resources
Charaxinae	Charaxini	*Polyura nepenthes*	15,333	KF990128	This study
Calinaginae	**-**	*Calinaga davidis*	15,267	HQ658143	Xia et al., 2011
Pseudergolinae	**-**	*Dichorragia nesimachus*	14,367	KF990126	This study
Satyrinae	Melanitini	*Melanitis Leda*	15,122	JF905446	Shi et al., 2013a
	Satyrini	*Eumenis autonoe*	15,489	GQ868707	Kim et al., 2010
	Satyrini	*Callerebia suroia*	15,208	KF906483	Shi et al., 2014
	Elymniini	*Elymnias hypermnestra*	15,167	KF906484	This study
	Elymniini	*Lethe dura*	15,259	KF906485	This study
Morphinae	**-**	*Stichophthalma howqua*	14,020	KF990129	This study
Heliconiinae	Argynnini	*Argyreus hyperbius*	15,156	JF439070	Wang et al., 2011
	Argynnini	*Fabriciana nerippe*	15,140	JF504707	Kim et al., 2011a
	Argynnini	*Issoria lathonia*	15,172	NC_018030	Unpublished
	Heliconiini	*Heliconius melpomene*	15,325	HE579083	Dasmahapatra et al., 2012
	Acraeini	*Acraea issoria*	15,245	GQ376195	Hu et al., 2010
	Acraeini	*Cethosia biblis*	15,211	KF990124	This study
Limenitidinae	Limenitidini	*Parathyma sulpitia*	15,268	JQ347260	Tian et al., 2012
Apaturinae	**-**	*Apatura ilia*	15,242	JF437925	Chen et al., 2012
	**-**	*Apatura metis*	15,236	NC_015537	Zhang et al., 2012
	**-**	*Sasakia charonda*	15,244	NC_014224	Wang et al., 2012
	**-**	*Sasakia funebris*	15,233	JX131328	Wang et al., 2013
	**-**	*Timelaea maculata*	15,178	KC572131	Cao et al., 2013
Biblidinae	**-**	*Ariadne ariadne*	15,179	KF990123	This study
Cyrestinae	**-**	*Cyrestis thyodamas*	15,254	KF990125	This study
Nymphalinae	Kallimini	*Kallima inachus*	15183	JN857943	Qin et al., 2012
	Melitaeini	*Melitaea cinxia*	15,170	NC_018029	Unpublished
	Junoniini	*Junonia orithya*	15,214	KF199862	Shi et al., 2013b
	Junoniini	*Hypolimnas bolina*	15,260	KF990127	This study
Libytheinae	**-**	*Libythea celtis*	15,164	NC_016724	Hao et al., 2013
Danainae	Danaini	*Euploea mulciber*	15,166	NC_016720	Hao et al., 2013
	Danaini	*Danaus plexippus*	15,314	NC_021452	Servín and Martínez, 2013
	Danaini	*Danaus chrysippus*	15,236	KF690637	Gan et al., 2014a
	Danaini	*Ideopsis similes*	15,200	KJ476729	Gan et al., 2014b
	Danaini	*Parantica aglea*	15,210	KM018020	This study
outgroup	Theclini	*Coreana raphaelis*	15,314	NC_007976	Kim et al., 2006
	Theclini	*Protantigius superans*	15,248	NC_016016	Kim et al., 2011b
	**-**	*Spindasis takanonis*	15,349	NC_016018	Kim et al., 2011b

The phylogenetic trees were reconstructed with the neighbor joining (NJ), maximum parsimony (MP), maximum likelihood (ML) and Bayesian inference (BI) methods based on two different nucleotide datasets as follows: D1 (13 PCGs only) and D2 (13 PCGs plus 2 rRNAs). The mitochondrial genes were separately aligned by MUSCLE in MEGA 5.0 software [32]. The PCGs were aligned according to their amino sequence similarity, whereas rRNAs were directly aligned according to sequence similarity using default settings. Then each of individual alignments was concatenated as a combined matrix. However, the scattergrams analysis showed that the substitution for third codon position of 13 PCGs trends toward saturation, potentially obscuring phylogenetic signals. Therefore, all third codon positions were excluded during the phylogenetic analysis. The NJ analysis was performed in the MEGA5.0 [32], the MP and ML analyses were carried out using the PAUP*4.0b10 [38], and the BI analysis was conducted using MrBayes 3.1.2 [39]. In the NJ analysis, nucleotide substitution model of the Kimura-2 parameter (K2P) was selected, and the bootstrap proportion values (BPs) with internal branch tests were obtained by 1,000 replicates to estimate the support levels for the nodes in the resultant topologies. The MP tree was reconstructed with branch swapping tree bisection-reconnection (TBR) heuristic search method, and the BPs were obtained after 1000 replicates by using 10 replicates of random stepwise additions of taxa. For ML and BI analyses, the two datasets were further partitioned by 4 strategies considering gene region and codon position. For D1 dataset, the partitioning strategies (PSs) were set as (1) 13 PCGs each as a single gene partition (D1-P13), (2) two partitions, including 1st and 2nd codon position of the PCGs (D1-P2). The PSs of D2 dataset were set as (3) 15 gene partitions (D2-P15) and (4) four partitions, including 1st and 2nd codon position of the PCGs and two rRNAs (D2-P4). The optimal substitution model of each partition was determined by Modeltest 3.7 [40], using the corrected Akaike information criterion (AICc). For ML analysis, the general time reversible model with invariant sites and among—site variation (GTR+I+G) was selected as the best fit model for each partition, and the BPs of the tree were also evaluated via the bootstrap test with 1000 iterations. The BI analysis was conducted with the same best fit substitution model used as the one selected for the ML analysis. Two independent runs of four incrementally heated MCMC chains (one cold chain and three hot chains) were simultaneously run for one million generations in all datasets, with each sampling of 100 generations, when the convergence of MCMC chains was achieved (<0.01), the first 25% of the sampled trees were discarded as samples of burn-in. The confidence values of the BI tree are presented as the Bayesian posterior probabilities (BPP). All the tree topologies were evaluated using the Approximately Unbiased (AU) [41], Shimodaira-Hasegawa (SH) [42], Kishino-Hasegawa (KH) [43], weighted SH (WSH) [42] and weighted KH (WKH) [43] methods by the Consel v0.2 software [44].

## Results and Discussion

### Genome structure and organization

**Fig 1:**
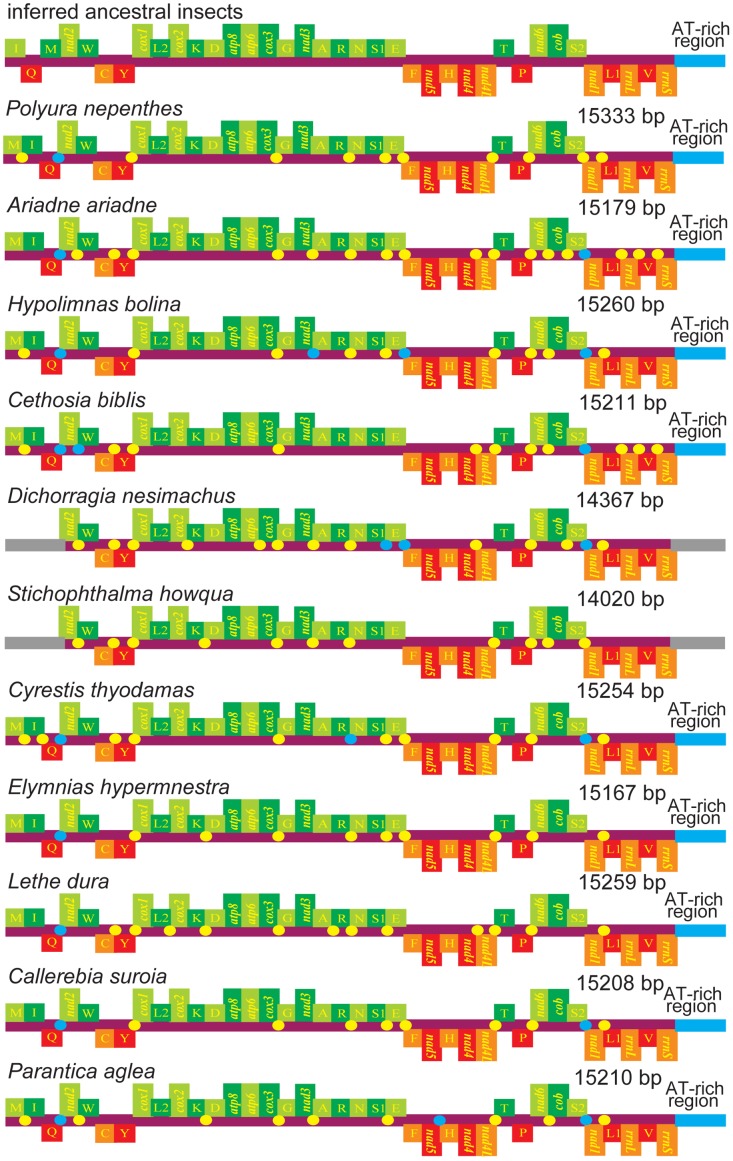
Gene arrangement of eleven nymphalid mitochondrial genomes sequenced in this study. Abbreviations for the genes are as follows: *cox1*, *cox2*, and *cox3* refer to the cytochrome oxidase subunits, *cob* refers to cytochrome b, and *nad1*—*nad6* refer to NADH dehydrogenase components, *rrnL* and *rrnS* refer to ribosomal RNAs. Transfer RNA genes are denoted by one letter symbols according to the IPUC-IUB one-letter amino acid codes. L1, L2, S1, S2 denote tRNA^Leu(CUN)^, tRNA^Leu(UUR)^, tRNA^Ser(AGN)^, tRNA^Ser(UCN)^, respectively. Genes coded on the majority strand are light/dark-green. Genes coded on the minority strand are red or orange. Alternation of colors was applied for distinction. The non-coding regions are presented as cyan/yellow dots. The unknown portions of partial mtDNAs are gray.

In this study, nine complete mitogenome sequences from *P*. *nepenthes* (15,333 bp), *A*. *ariadne* (15,179 bp), *H*. *bolina* (15,260 bp), *C*. *biblis* (15,211 bp), *E*. *hypermnestra* (15,167 bp), *L*. *dura* (15,259 bp), *C*. *suroia* (15,208 bp), *C*. *thyodamas* (15,254 bp), *P*. *aglea* (15,210 bp) and two nearly complete mitogenomes from *D*. *nesimachus* (14,367 bp) and *S*. *howqua* (14,020 bp) were determined, representing nine subfamilies of Nymphalidae (Tables 1 and 2). All genes identified in the eleven mitochondrial genomes are typical insect mitochondrial genes with normal gene sizes [45]. In all, 37 genes (13 PCGs, 22 tRNAs, 2 rRNAs) and an A+T-rich region were identified in the nine completely sequenced mitogenomes, in which, the gene order was identical to that of the other nymphalid mitogenomes sequenced to date, but different from the gene order in inferred ancestral insects [45] (Fig 1). The regions that we failed to sequence in other two mitogenomes were A+T-rich region and gene cluster *trnM*—*trnI*—*trnQ*, which are usually located in or around *rrnS* and *nad2* (Fig 1), where extremely high A+T content and stable stem-loop structures may have disrupted PCR and sequencing reactions, a common problem in sequencing insect mitochondrial genomes [23, 46].

**Table 2:** Characterization of the nymphalid mitogenomes used in this study.

Species	Whole genome		PCG^a^		*rrnL*		*rrnS*		AT-rich region	
	Size (bp)	A+T (%)	Size (bp)^b^	A+T (%)	Size (bp)	A+T (%)	Size (bp)	A+T (%)	Size (bp)	A+T (%)
*Apatura ilia*	15,242	80.5	11,133	78.9	1,333	84.0	776	84.9	403	92.5
*Apatura metis*	15,236	80.5	11,109	78.9	1,333	84.5	779	84.8	394	92.9
*Sasakia charonda*	15,244	79.9	11,108	78.2	1,323	84.4	775	85.0	380	91.8
*Sasakia funebris*	15,233	81.2	11,181	80.0	1,333	84.6	772	85.5	370	93.0
*Timelaea maculate*	15,178	81.1	11,145	79.6	1,332	85.0	777	85.9	382	93.2
*Ariadne ariadne*	15,179	80.1	11,163	78.8	1,320	83.9	799	84.1	319	92.8
*Cyrestis thyodamas*	15,254	81.1	11,181	79.7	1,328	84.6	780	85.1	380	91.6
*Kallima inachus*	15,183	80.3	11,163	79.2	1,335	82.7	774	85.1	376	92.0
*Melitaea cinxia*	15,170	80.0	11,154	78.6	1,336	84.6	772	84.6	338	92.9
*Hypolimnas bolina*	15,260	79.7	11,154	78.1	1,333	83.3	776	84.9	356	93.3
*Junonia orithya*	15,214	80.4	11,154	79.2	1,326	82.7	775	84.9	331	94.9
*Dichorragia nesimachus* ^▲^	14,367	81.1	11,001	80.3	1,369	85.0	621	83.3	-	-
*Argyreus hyperbius*	15,156	80.8	11,154	79.5	1,330	84.5	778	85.2	349	95.4
*Acraea issoria*	15,245	79.7	11,151	78.0	1,331	83.9	788	83.7	430	96.0
*Fabriciana nerippe*	15,140	80.9	11,157	79.6	1,321	84.4	773	84.9	329	95.4
*Issoria lathonia*	15,172	81.1	11,154	79.9	1,319	84.4	771	85.1	361	96.1
*Heliconius melpomene*	15,328	81.7	11,106	80.3	1,364	85.7	779	85.1	268	95.9
*Cethosia biblis*	15,211	79.8	11,163	78.4	1,316	83.7	774	85.5	334	93.4
*Parathyma sulpitia*	15,268	81.9	11,187	80.6	1,319	84.7	779	85.7	349	94.6
*Libythea celtis*	15,164	81.2	11,166	80.0	1,335	84.7	775	85.4	328	96.3
*Eumenis autonoe*	15,489	79.1	11,184	76.8	1,335	83.7	775	85.3	678	94.5
*Melanitis leda*	15,122	79.8	11,163	78.4	1,332	84.0	774	85.0	314	89.5
*Elymnias hypermnestra*	15,167	80.4	11,160	79.1	1,331	84.7	768	85.0	405	90.6
*Lethe dura*	15,259	79.3	11,181	77.5	1,341	83.8	775	85.7	409	92.2
*Callerebia suroia*	15,208	79.5	11,121	77.9	1,345	84.3	777	85.7	393	94.2
*Stichophthalma howqua* ^▲^	14,020	78.5	10,848	77.0	1,332	84.6	600	84.2	-	-
*Polyura nepenthes*	15,333	80.8	11,214	79.4	1,389	84.9	772	84.5	437	89.5
*Calinaga davidis*	15,267	80.4	11,211	78.8	1,337	83.8	773	85.9	389	92.0
*Euploea mulciber*	15,166	81.5	11,139	80.2	1,314	84.6	776	85.3	399	93.5
*Danaus plexippus*	15,314	81.3	11,148	79.9	1,340	84.4	774	86.1	469	94.5
*Danaus chrysippus*	15,236	80.3	11,145	78.7	1,314	83.8	779	84.7	418	95.0
*Ideopsis similes*	15,200	81.6	11,121	80.2	1,315	84.7	776	85.9	404	95.7
*Parantica aglea*	15,210	79.6	11,121	77.8	1,309	84.1	775	84.9	443	93.2

^▲^ Partial mitogenome lacking in the A+T-rich region, *trnM*, *trnI* and *trnQ*, partial *nad2* and *rrnS* sequence. Bar (-) indicates lack of sequence information for the A + T region in the genome.

^a^ Protein coding genes.

^b^ Termination codons were excluded in total size count.

All analyzed nymphalid mitogenomes are consistently AT biased, with values from 79.1% in *Eumenis autonoe* (subfamily Satyrinae) to 81.9% in *Parathyma sulpitia* (subfamily Limenitidinae), averaging at 80.5% (Table 2). However, these values fall within the range of the A+T content for other Lepidoptera species, from 77.8% in *Ochrogaster lunifer* [47] to 82.7% in *C*. *raphaelis* [48]. The nucleotide skewness statistics for all nymphalid mitogenomes indicate slight A or T skews with AT-skew values ranging from -0.068 in *C*. *biblis* (subfamily Heliconiinae) to 0.019 in *H*. *bolina* (subfamily Nymphalinae), and moderate C skews with GC-skew values ranging from -0.274 in *S*. *howqua* (subfamily Morphinae) to -0.178 in *P*. *Sulpitia* (subfamily Limenitidinae) (S1 Fig). A similar trend has been observed in other lepidopterans, the AT-skew of which ranges from -0.047 (*C*. *raphaelis*) to 0.059 (*Bombyx mori*) and the GC-skew from -0.318 (*O*. *lunifer*) to -0.158 (*C*. *raphaelis*) [47, 48].

### Protein coding genes

Like those of other determined lepidopteran mitogenomes, nine PCGs (*nad2*, *cox1*, *cox2*, *atp8*, *atp6*, *cox3*, *nad3*, *nad6*, and *cob*) of the eleven mitogenomes are coded on the majority strand, while the other four genes (*nad1*, *nad4*, *nad4L*, *nad5*) coded on the minority strand. All PCGs are initiated by typical ATN, with the exception of the *cox1* which uses the unusual CGA(R) as observed in most other sequenced lepidopterans [35, 47, 49–54]; all PCGs are terminated with TAA/TAG, or with truncated codons TA or T which are presumed to be completed via post-transcriptional polyadenylation [55, 56] (S2 Table). The A+T contents of the PCGs, excluding stop codons, were similar to other nymphalid mitogenomes, and fall within the range from 76.8% in *E*. *autonoe* (subfamily Satyrinae) to 80.6% in *P*. *sulpitia* (subfamily Limenitidinae), with the average value equal to 79.0%. In all newly sequenced mitogenomes, the third codon positions have a considerably higher A+T content than the first and second positions (S2 Fig) as seen in other sequenced nymphalids.

**Fig 2:**
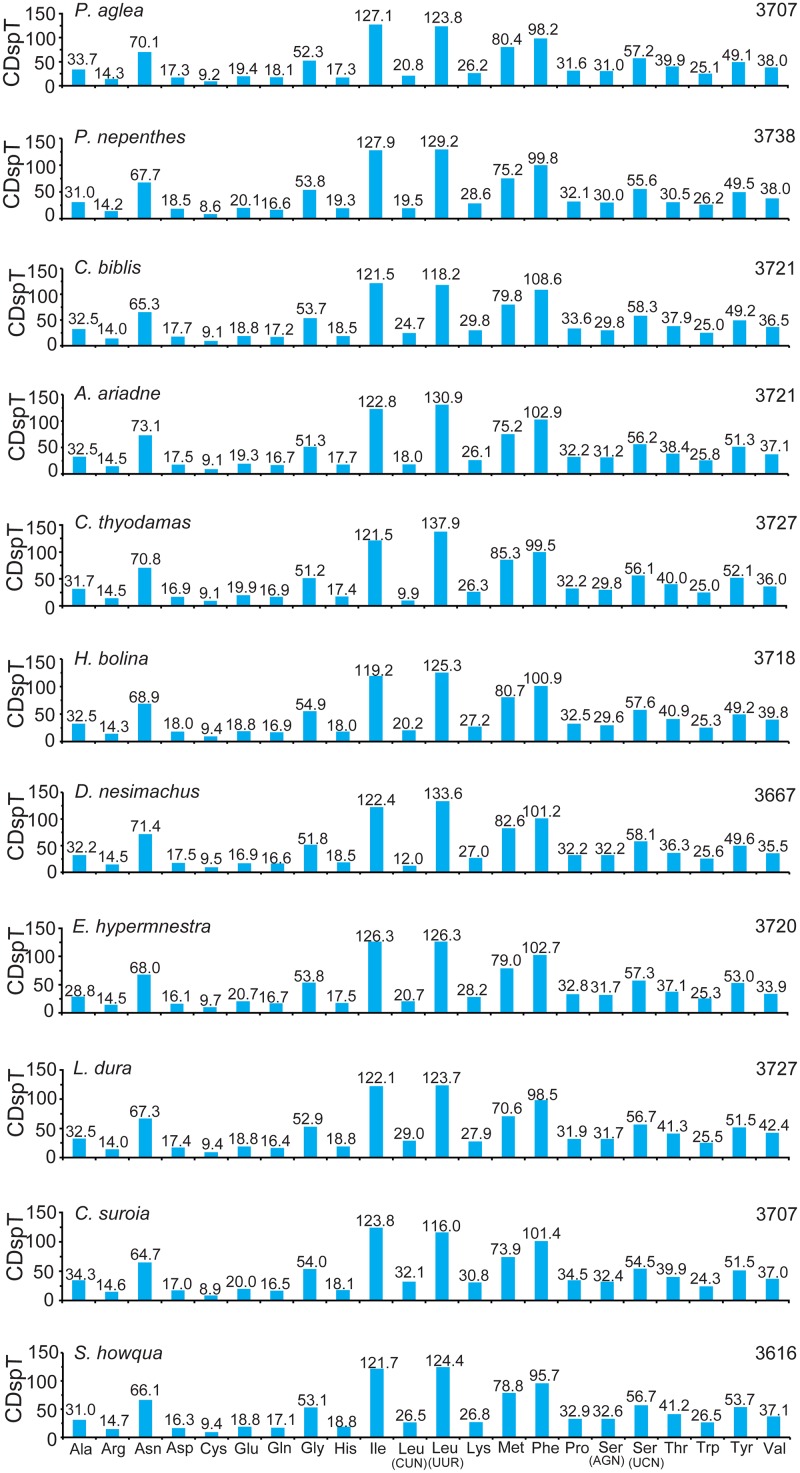
Codon distribution in the eleven newly sequenced nymphalid mitogenomes. Numbers to the left refer to the total number of codons. CDspT, codons per thousand codons. Codon families are provided on the x axis.

**Fig 3:**
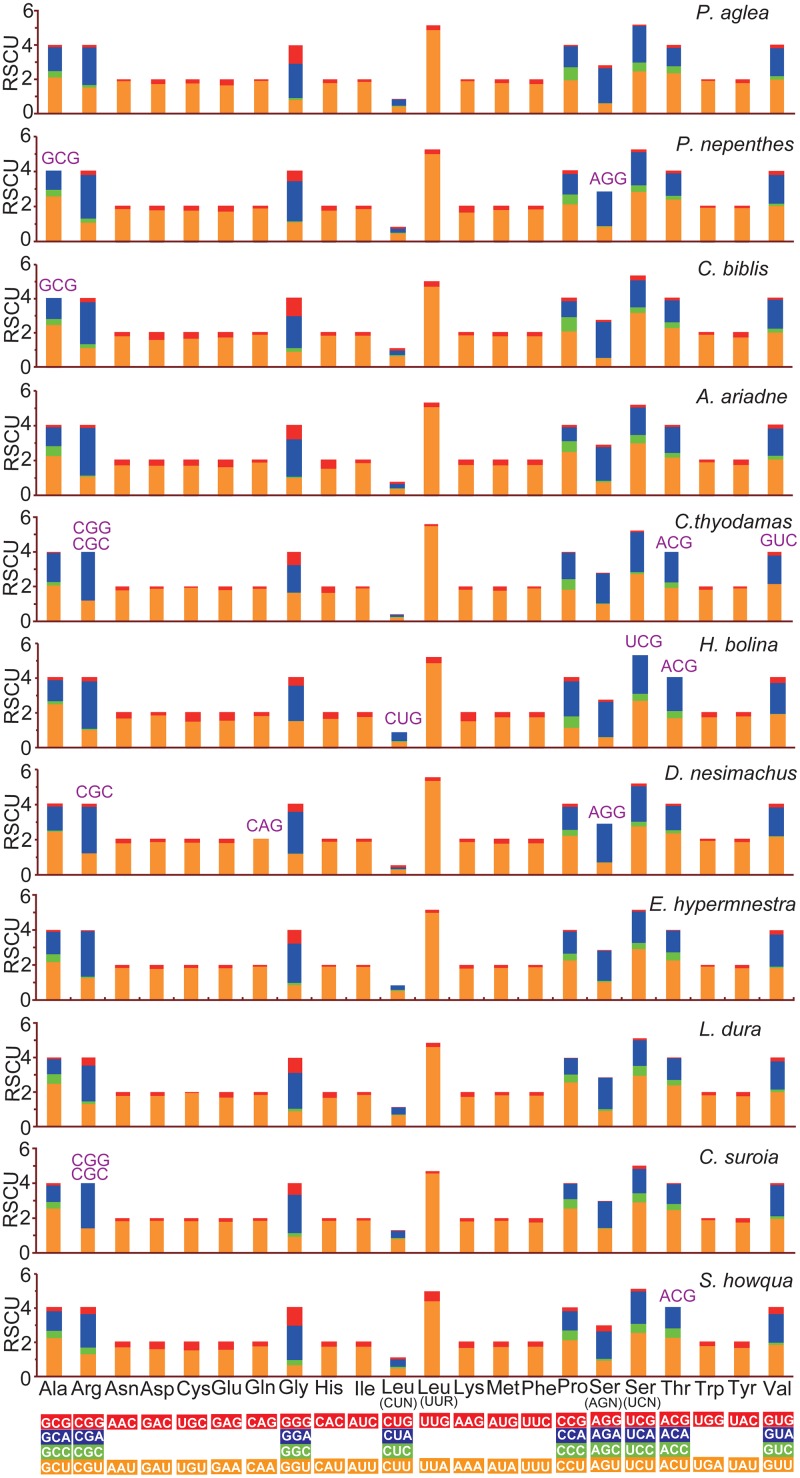
Relative synonymous codon usage (RSCU) in the eleven newly sequenced nymphalid mitogenomes. Codon families are given on the x axis. Codons that are not present in the genome are indicated in purple.

The abundance of codon families and Relative Synonymous Codon Usage (RSCU) in 13 PCGs were investigated for the eleven newly determined mitogenomes, as shown in Figs 2 and 3. All stop codons, complete and incomplete, were excluded from the analysis to avoid biases due to incomplete stop codons. Total number of non-stop codons (CDs) in nine newly sequenced complete mitogenomes is very similar to that in other available nymphalid mitogenomes, ranging from 3,695 of *S*. *charonda* (subfamily Apaturinae) to 3,738 of *Polyura nepenthes* (subfamily Charaxinae) (S3 Fig). Although the numbers of CDs in two nearly complete mitogenomes are less than that in other complete mitogenomes, the codon families exhibit a very similar behavior among all newly sequenced mitogenomes. The five most frequently used codon families (*Leu (UUR)*, *Ile*, *Phe*, *Met* and *Asn*), each with at least 60 CDs per thousand CDs, were two-fold degenerate in codon usage and were rich in A and T in every mitogenome. The three codon families with at least 100 CDs per thousand (*Leu (UUR)*, *Ile* and *Phe*) were found in the *C*. *biblis* (subfamily Heliconiinae), *A*. *ariadne* (subfamily Biblidinae), *H*. *bolina* (subfamily Nymphalinae), *D*. *nesimachus* (subfamily Pseudergolinae), *E*. *hypermnestra* and *C*. *suroia* (subfamily Satyrinae) mitogenomes, whereas only *Leu (UUR)* and *Ile* units were found in remaining five newly sequenced mitogenomes. However, the rarest used codon family is Cys, which displayed no more than 10 CDs per thousand in eleven mitogenomes (Fig 2). Additionally, the RSCU values of NNU and NNA are greater than 1 (excluding the NNA for *Leu (UUR)*), indicating a strong A+T-bias in their third codon position, whereas the lost codons usually belong to G+C-rich codons (Fig 3). The codon usage bias may be positively correlated with the AT bias of the third codon position for the insect mitogenomes [47, 49, 52, 57–60].

### Transfer RNA and ribosomal RNA genes

All tRNAs of the eleven nymphalid species harbor the typical cloverleaf structures commonly found in insects, except for *trnS1(AGN)* whose dihydrouridine (DHU) arm is replaced by a simple loop (maps of secondary structure are not shown here). Two rRNA (*rrnL* and *rrnS*) genes are located between *trnL1 (CUN)* and *trnV*, and between *trnV* and the A+T-rich region, respectively (Fig 1). Of these two rRNAs, the larger one (*rrnL* gene) has attracted much more attention in the studies of systematic evolution and classification [61]. Here, we first compared the entire secondary structure of all the nymphalid *rrnL* genes for more phylogenetically informative signals. The results showed that all the *rrnL* genes harbored six domains (domain III is absent in Arthropoda) with 49 helices, which is broadly congruent with those proposed for other insects [46, 47]. *D*. *nesimachus* was shown in S4 Fig as an example with the remaining species not listed. Among this nymphalid species, some of the *rrnL* highly variable regions, such as the stem-loops of H837/D13, 14 in domain II and H2077/G3 in domain V were also shown (S5 and S6 Figs). The structural characteristics of H837/D13, 14 and H2077/G3, suggest that the *A*. *ariadne* (subfamily Biblidinae) is closer with *C*. *thyodamas* (subfamily Cyrestinae) than with *D*. *nesimachus* (subfamily Pseudergolinae), the *S*. *howqua* (subfamily Morphinae) is most likely to be related to Satyrinae species, and so on.

### Non-coding regions

The non-coding (NC) regions of eleven newly sequenced mitogenomes were illustrated in Fig 1. There were three distinct large NC regions with at least 15 bp in all complete mitogenomes. The first large NC region located between *trnQ* and *nad2*, appeared to be common in the nine mitogenomes (including *P*. *nepenthes*, *A*. *ariadne*, *H*. *bolina*, *C*. *biblis*, *E*. *hypermnestra*, *L*. *dura*, *C*. *suroia*, *C*. *thyodamas*, *P*. *aglea*), and this region was also detected in other nymphalid mitogenomes, varying in length from 40 bp in *E*. *hypermnestra* (subfamily Satyrinae) to 87 bp in *S*. *charonda* and *Sasakia funebris* (subfamily Apaturinae). The second large NC region inserted between *trnS2* and *nad1*, was present in *A*. *ariadne* (subfamily Biblidinae), *H*. *bolina* (subfamily Nymphalinae), *C*. *biblis* (subfamily Heliconiinae), *C*. *thyodamas* (subfamily Cyrestinae), *C*. *suroia* (subfamily Satyrinae) and *P*. *aglea* (subfamily Danainae) mitogenomes, whereas this region was absent in *E*. *hypermnestra* and *L*. *dura* (subfamily Satyrinae) mitogenomes or showed no more than 15 bp in *P*. *nepenthes* (subfamily Charaxinae) mitogenome. The third large NC region located between *rrnS* and *trnM*, coincided with the A+T-rich region, also called the CR. The CR regions have no conspicuous macro-repeat units (>50 bp), which are occasionally found in other lepidopteran insects [62–65]. Nonetheless, several conserved structures characteristic of lepidopterans are observed, such as the poly-T stretch preceded by the ATAGA motif neighboring the *rrnS* gene, and the microsatellite-like elements (TA)n (n = 7–9) preceded by the ATTTA motif. Although the CR regions were not sequenced in two incomplete mitogenomes (*D*. *nesimachus* and *S*. *howqua*), a few long NC regions (>15 bp in length), including the region located between *trnS2* and *nad1*, were also observed in *D*. *nesimachus* (subfamily Pseudergolinae) mitogenome, however, the similar region was not found in *S*. *howqua* (subfamily Morphinae) mitogenome.

### Phylogenetic analysis

**Fig 4:**
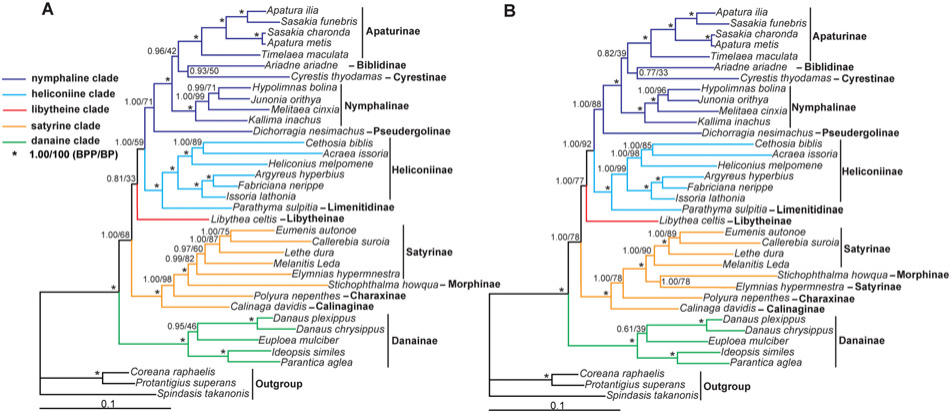
Phylogenetic relationships among 33 nymphalid species. (A) Bayesian Inference and maximum likelihood phylogram obtained with the D1 dataset, which is divided into 13 partitions (D1-P13). (B) Bayesian Inference and maximum likelihood phylogram obtained with the D2 dataset, which is divided into 15 partitions (D2-P14). Numbers on each node correspond to the posterior probability values of the BI analysis (left) and the ML bootstrap percentage values for 1 000 replicates of ML analysis (right).

**Fig 5:**
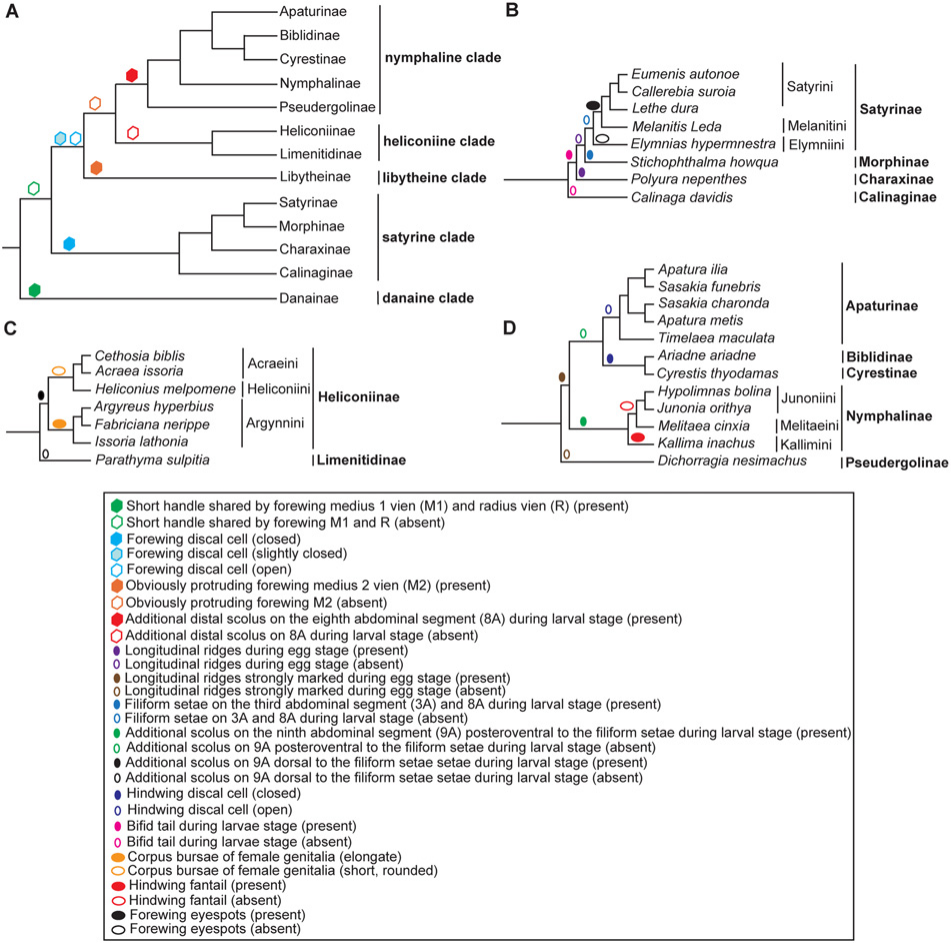
Morphological character distribution mapped on the mitogenomic tree of Nymphalidae and internal clades (All the morphological characters selected are taken after Freitas and Brown, 2004). (A) the topology of Nymphalidae clades; (B) the topology of satyrine clade; (C) the topology of heliconiine clade; (D) the topology of nymphaline clade.

The phylogenetic reconstructions conducted in this study using multiple methods have produced generally similar topology for most of the major nymphalid groups. The phylogenetic relationships inferred by BI and ML methods were highly congruent and strongly supported (BPP >0.95 and BP >85% for most nodes), across the partitions for each of the two datasets (Fig 4). The topology tests show that the BI trees from PS1 (BI-P13 tree) (Fig 4A), PS2 (BI-P2 tree) (not shown), PS3 (BI-P15 tree) (Fig 4B) and PS4 (BI-P4 tree) (not shown) are the best trees for the D1 and D2 dataset (Table 3). All the trees reveal that the Nymphalidae include five major clades, namely, the nymphaline clade (including five subfamilies: Apaturinae, Biblidinae, Cyrestinae, Nymphalinae and Pseudergolinae); heliconiine clade (including two subfamilies: Heliconiinae and Limenitidinae); satyrine clade (including four subfamilies: Satyrinae, Morphinae, Charaxinae and Calinaginae); danaine clade (including single subfamily: Danainae); libytheine clade (including one subfamily: Libytheinae). These results are consistent with those obtained from previous molecular studies [5, 14, 66], whereas remarkably different from the results of morphology-based phylogenetic studies, such as the cladistic analysis by Freitas and Brown [15] based on 234 characters of different developmental stages. In order to further evaluate the probable congruence between molecular and morphological evolutions, we carefully examined all the morphological characters from Freitas and Brown [15] and others and tried to map some of them on the mitogenomic phylogenetic trees robustly obtained in this study (Fig 5), indicating that some of the selected morphological characters (developmental and adult) are somewhat compatible with the molecular phylogeny. For example, the danaine as the basal group in Nymphalidae is supported by a unique morphological plesiomorphy, the connected medius 1 vein (M1) and radius vein (R), whereas the remaining nymphalid groups lack the morphological character (Fig 5A, green hexagon); the satyrine is characterized by a closed discal cell of the forewing, whereas its sister groups (libytheine + heliconiine + nymphaline) have a slightly closed or open discal cell of the forewing (Fig 5A, cyan hexagon).

**Table 3:** Topological tests for two datasets with partitions.

datasets	trees	statistical tests*					
		obs	AU	KH	SH	WKH	WSH
D1	BI-P13/P2	0.0	0.821	0.000	0.838	0.000	0.838
	ML-P13/P2	0.0	0.454	0.318	0.763	0.318	0.700
	NJ	37.7	0.097	0.085	0.124	0.085	0.138
	MP	38.9	0.068	0.055	0.113	0.055	0.099
D2	BI-P15/P4	0.0	0.629	0.522	0.767	0.522	0.770
	ML-P15/P4	0.0	0.552	0.478	0.651	0.478	0.800
	MP	0.5	0.551	0.477	0.651	0.041	0.525
	NJ	15.4	0.197	0.198	0.278	0.198	0.257

* obs: the log-likelihood difference δ_x_ to the best tree; AU = Approximately Unbiased; KH = Kishino-Hasegawa; SH = Shimodaira-Hasegawa; WKH = weighted KH; WSH = weighted SH.

The danaids (danaine clade), formerly classified as a family under the order Lepidoptera, are now treated as a subfamily of Nymphalidae. Our study indicates that Danainae (represented by *Euploea mulciber*, *Danaus plexippus*, *Danaus chrysippus*, *Ideopsis similes*, *P*. *aglea*) is sister to the remaining nymphalids including the libytheines with strong supports (BPP = 1.00 in two BI trees and BP = 100% in two ML trees, respectively), same as results from other recent analyses [54, 66–68]. Additionally, the analysis of mitochondrial *rrnL* secondary structures by Hao et al. [69] showed that the *rrnL* morphological characteristics of the danaid species were markedly different from other nymphalid groups. Morphologically, the danaids possess some of their unique features, such as the connected M1 vein and R vein in the forewing, which are markedly distinct from other nymphalid butterflies.

The satyrine clade includes Satyrinae, Morphinae, Charaxinae and Calinaginae, compatible with results of some previous studies based on morphological [15], molecular [10, 14, 70], or combined data [5, 18]. The Calinaginae (represented by *Calinaga davidis*) is the basal group of entire satyrine clade, instead of the sister relationship to Charaxinae as previously stated [14]. The Charaxinae (represented by *P*. *nepenthes*) is the sister to the grouping of Satyrinae plus Morphinae (Fig 3), consistent with those of multiple-gene based study [20] and most comprehensive taxa-sampling studies [5]. The association of Charaxinae with Satyrinae plus Morphinae is also supported by the morphological features, for example, all groups of satyrine clade, excluding the subfamily Calinaginae, have a bifid tail during their larval stages (Fig 5B). However, the phylogenetic position of Morphinae was not stable among trees from different dataset (Fig 4). Morphologically, the Morphinae has some distinct characteristics, such as the filiform setae on the third abdominal segment (3A) and the eighth abdominal segment (8A) during the larval stage [15] (Fig 5B). In addition, the Amathusiini and Elymniini occupy the same host plant (Arecaceae) during the larval stages, implying a close relationship. It has been proposed that Morphinae (Morphini, Brassolini and Amathusiini) should be grouped within Satyrinae [5, 20, 70–72], though other studies suggested two independent subfamilies [12, 14].

The phylogenetic status of libytheines (libytheine clade) within nymphalids has long standed as a controversial issue. They were traditionally treated as a separate family owing to their unusual morphological features such as their exceptionally long labial palpus and fully developed forelegs in female [73–75]. However, some scholars proposed that libytheines should be included in Nymphalidae as a subfamily for the presence of longitudinal ridges on the antenna shared with Nymphalidae [12, 76], sister to all other Nymphalidae because of the lack of apomorphic features such as the simple female foreleg [7, 8, 15]. Evidences from host plant use and geographic distribution together suggest that this group is sister to the remaining Nymphalidae, as a basal subfamily [9, 77, 78], and some recent studies also suggested Libytheinae or the grouping of Libytheinae plus Danainae was the basal lineage of the Nymphalidae in molecular or molecular plus morphological view [5, 10, 14, 20, 79]. In this study, *L*. *celtis* (subfamily Libytheinae) was revealed as the sister of the grouping of nymphaline plus heliconiine clades in all phylogenetic trees with strong supports, except the ML-P13 tree (33% BP value at the node) (Fig 4A). This result was concordant with previous mitogenomic phylogenies [67, 68]. Thus, the phylogenetic position of Libytheinae remains uncertain.

The heliconiine clade comprises representatives of the subfamilies Heliconiinae and Limenitidinae. In this study, though our taxon sampling is limited, tribal-level relationships within subfamily Heliconiinae are well supported (Fig 4). The tribe Argynnini (represented by *Argyreus hyperbius*, *Fabriciana nerippe*, *Issoria lathonia*) is sister to the group composed of Acraeini (represented by *Acraea issoria*, *C*. *biblis*) + Heliconiini (represented by *Heliconius melpomene*) (BPP = 1.00 in two BI trees, BP = 100%, 99% in ML-P13 and ML-P15 trees respectively). This outcome is concordant with those reported earlier based on molecular data [5, 10, 14, 21, 80], but inconsistent with two morphologically-based studies [15, 81], in which Acraeini emerged as sister to the grouping of (Heliconiini + (Vagrantini + Argynnini)).

The final major clade, the nymphaline clade, contains representatives of the subfamilies Apaturinae, Biblidinae, Cyrestinae, Nymphalinae and Pseudergolinae. However, their internal relationships, especially regarding the Pseudergolinae, Biblidinae and Cyrestinae, are still controversial [5, 14, 20, 21]. In this study, the Pseudergolinae (reprented by *D*. *nesimachus*) is placed as sister to other groups of the nymphaline clade with strong or moderate support values (BPP = 1.0 in two BI trees, BP = 71%, 88% in ML-P13 and ML-P15 trees) (Fig 4), in concordance with the result of Wahlberg et al. [5]. The *C*. *thyodamas* (subfamily Cyrestinae) is the sister of *A*. *ariadne* (subfamily Biblidinae) with strong or moderate support (BPP = 0.93 in BI-P13 and BPP = 0.77 in BI-P15 tree), but weakly supported in ML-P13 tree (BP = 50%) and ML-P15 tree (BP = 33%) (Fig 4). This relationship is consistent with that by Zhang et al. [21], but contradicting that of Walhberg et al. [5] and Wahlberg and Wheat [20], which suggested that the Cyrestinae was sister to Nymphalinae. Morphologically, the Cyrestinae and Biblidinae are similar, as they share the short forewing discal cell, which is not found in other nymphalid groups. As for the Apaturinae, in this study, their five representatives (*Apatura ilia*, *Apatura metis*, *S*. *charonda*, *S*. *funebris*, *Timelaea maculata*) formed a strongly supported monophyletic group with the Biblidinae plus Cyrestinae as the sister group, albeit with weak support in ML-P13 tree (BP = 42%) and ML-P15 tree (BP = 39%). All of the three subfamilies were grouped with the Nymphalinae (represented by *Kallima inachus*, *Melitaea cinxia*, *H*. *bolina*, *Junonia orithya*) as its sister taxon, formerly revealed as the Heliconiinae by Freitas and Brown [15], Biblidinae + Apaturinae by Brower [10], Apaturinae by Wahlberg et al. [14] or Cyrestinae by Wahlberg et al. [5]. We found that the internal relationships of the nymphaline clade established in this study are consistent with their morphological pattern; e.g., all groups of nymphaline clade, excluding the basal group (Pseudergolinae), have a strongly marked longitudinal ridges during the egg stage; the Nymphalinae is characterized by an additional scolus on the ninth abdominal segment (9A) posteroventral to the filiform setae during the larval stage, which is absent in its sister groups ((Biblidinae + Cyrestinae) + Apaturinae) [15] (Fig 5D).

## Supporting Information

S1 Fig Scatter plot of AT- and GC-skews in the nymphalid subfamilies. Composition skewness was calculated according to the formulas (AT-skew = [A-T] / [A+T]; GC-skew = [G-C] / [G+C]). All the species that are represented are listed in Table 1.
(TIF)

S2 Fig The variation of AT content in different codon position of the concatenated 13 PCGs in the Nymphalidae mitogenomes. The 1st, 2nd and 3rd represent nucleotide codon position 1, 2 and 3 respectively. All the species that are represented are listed in Table 1.
(TIF)

S3 Fig Comparison of the total number of the amino acids of the mitogenomic PCGs across the nymphalid species. All the species that are represented are listed in Table 1.
(TIF)

S4 Fig Predicted *rrnL* secondary structure in *D*. *nesimachus* mitogenome. Roman numerals denote the conserved domain structure. Helices are shaded in grey. Tertiary structures are denoted by boxed bases joined by solid lines. Base-pairing is indicated as follows: Watson-Crick pairs by dashes, wobble GU pairs by red dots and other non-canonical pairs by green dots.
(TIF)

S5 Fig The secondary structures of H837/D13, 14 in *rrnL* gene from all Nymphalidae species used in this study. Symbols are as in S4 Fig.
(TIF)

S6 Fig The secondary structures of H2077/G3 in *rrnL* gene from all Nymphalidae species used in this study. Symbols are as in S4 Fig.
(TIF)

S1 Table Materials and their sources in this study. (PDF)

S2 Table Predicted initiation and termination codons distribution of the 13 mitochondrial PCGs among Nymphalidae species in this study. (PDF)
