# Supplementary figures and images for: Morphological Characters Are Compatible with Mitogenomic Data in Resolving the Phylogeny of Nymphalid Butterflies (Lepidoptera: Papilionoidea: Nymphalidae)
Source: PLoS One. 2015 Apr 10;10(4):e0124349. doi: 10.1371/journal.pone.0124349 (PMC4393276; doi:10.1371/journal.pone.0124349)

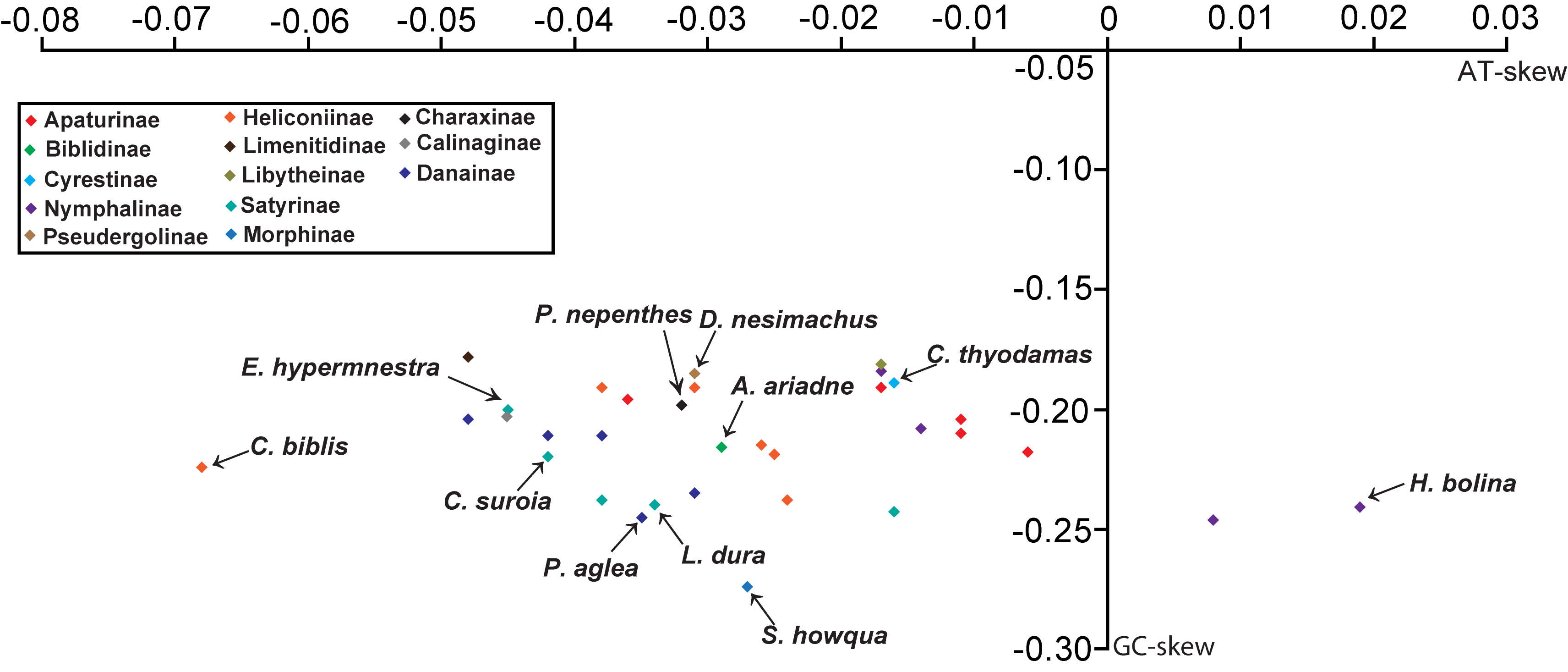

Supplement: S1 Fig — Composition skewness was calculated according to the formulas (AT-skew = [A-T] / [A+T]; GC-skew = [G-C] / [G+C]). All the species that are represented are listed in Table 1. (TIF) [file pone.0124349.s001.tif]

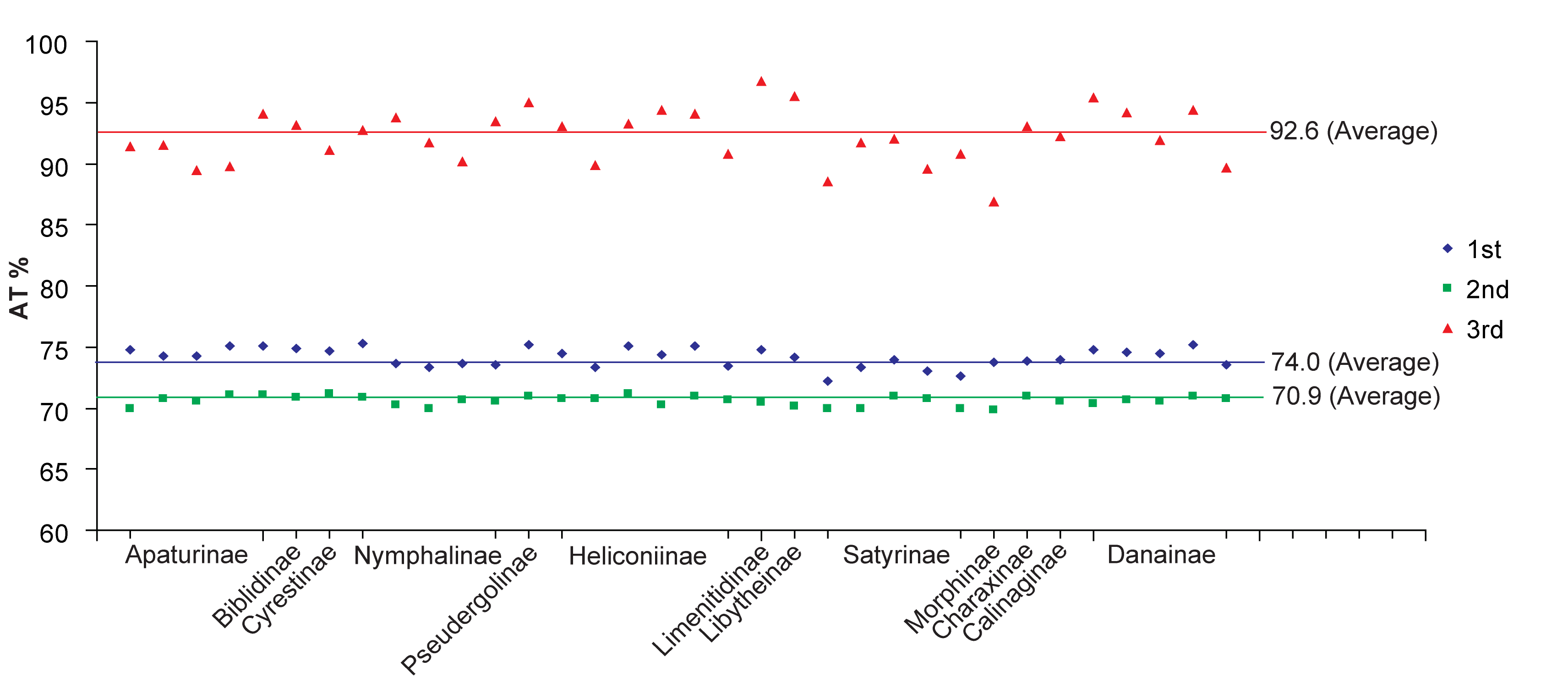

Supplement: S2 Fig — The 1st, 2nd and 3rd represent nucleotide codon position 1, 2 and 3 respectively. All the species that are represented are listed in Table 1. (TIF) [file pone.0124349.s002.tif]

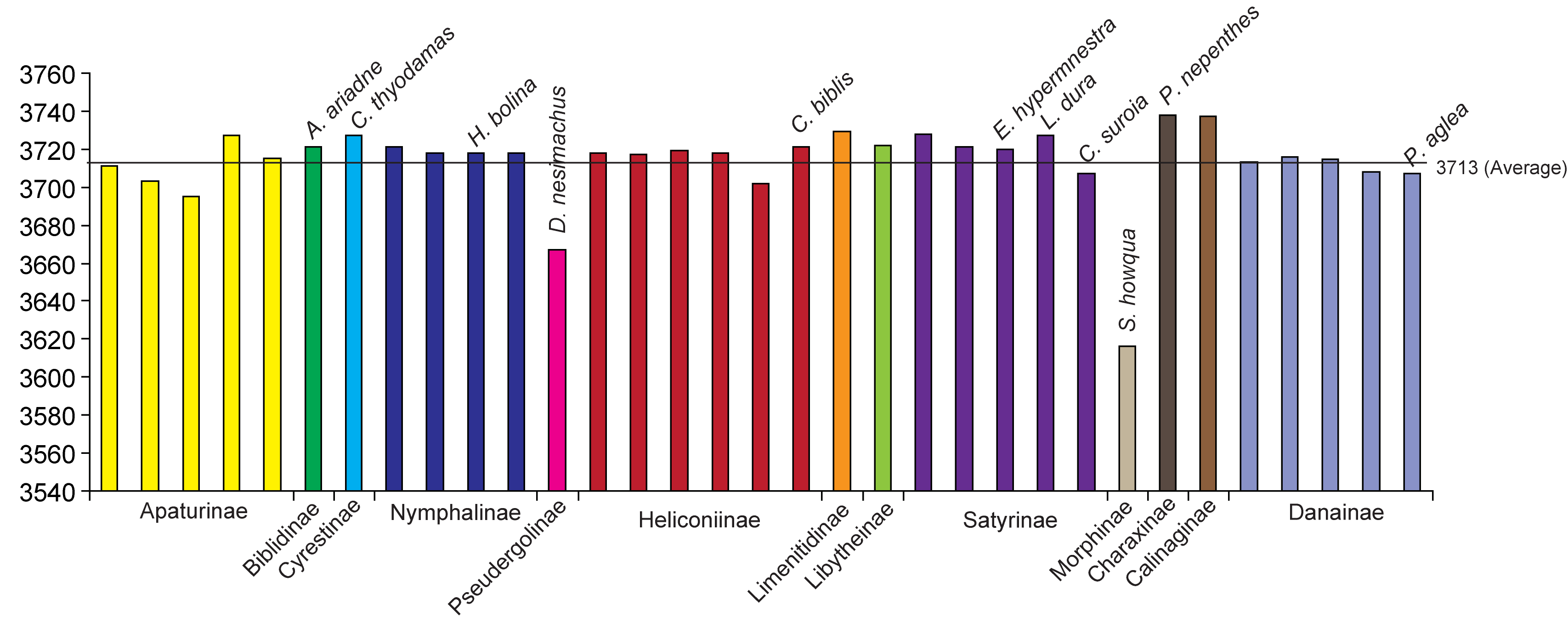

Supplement: S3 Fig — All the species that are represented are listed in Table 1. (TIF) [file pone.0124349.s003.tif]

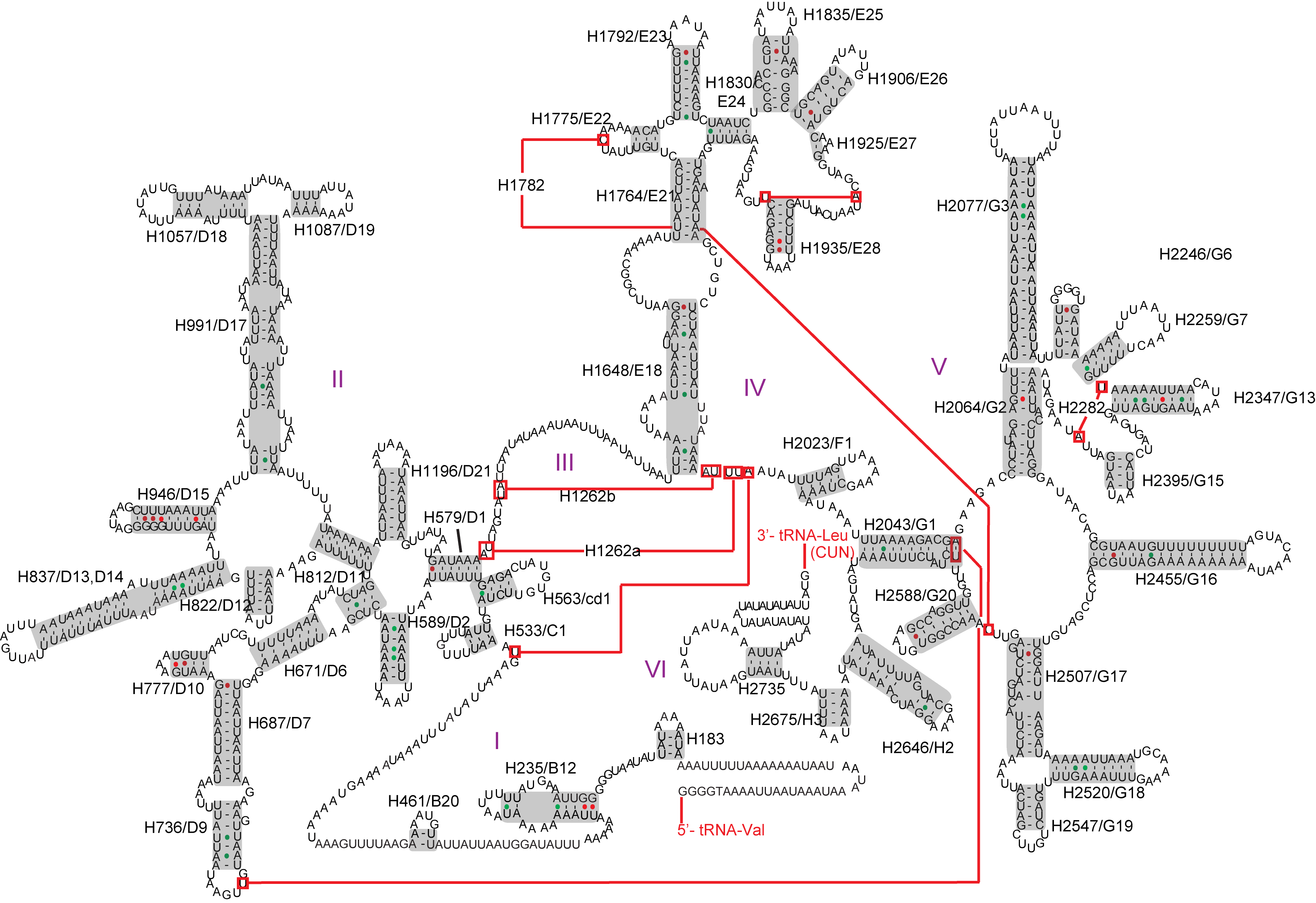

Supplement: S4 Fig — Roman numerals denote the conserved domain structure. Helices are shaded in grey. Tertiary structures are denoted by boxed bases joined by solid lines. Base-pairing is indicated as follows: Watson-Crick pairs by dashes, wobble GU pairs by red dots and other non-canonical pairs by green dots. (TIF) [file pone.0124349.s004.tif]

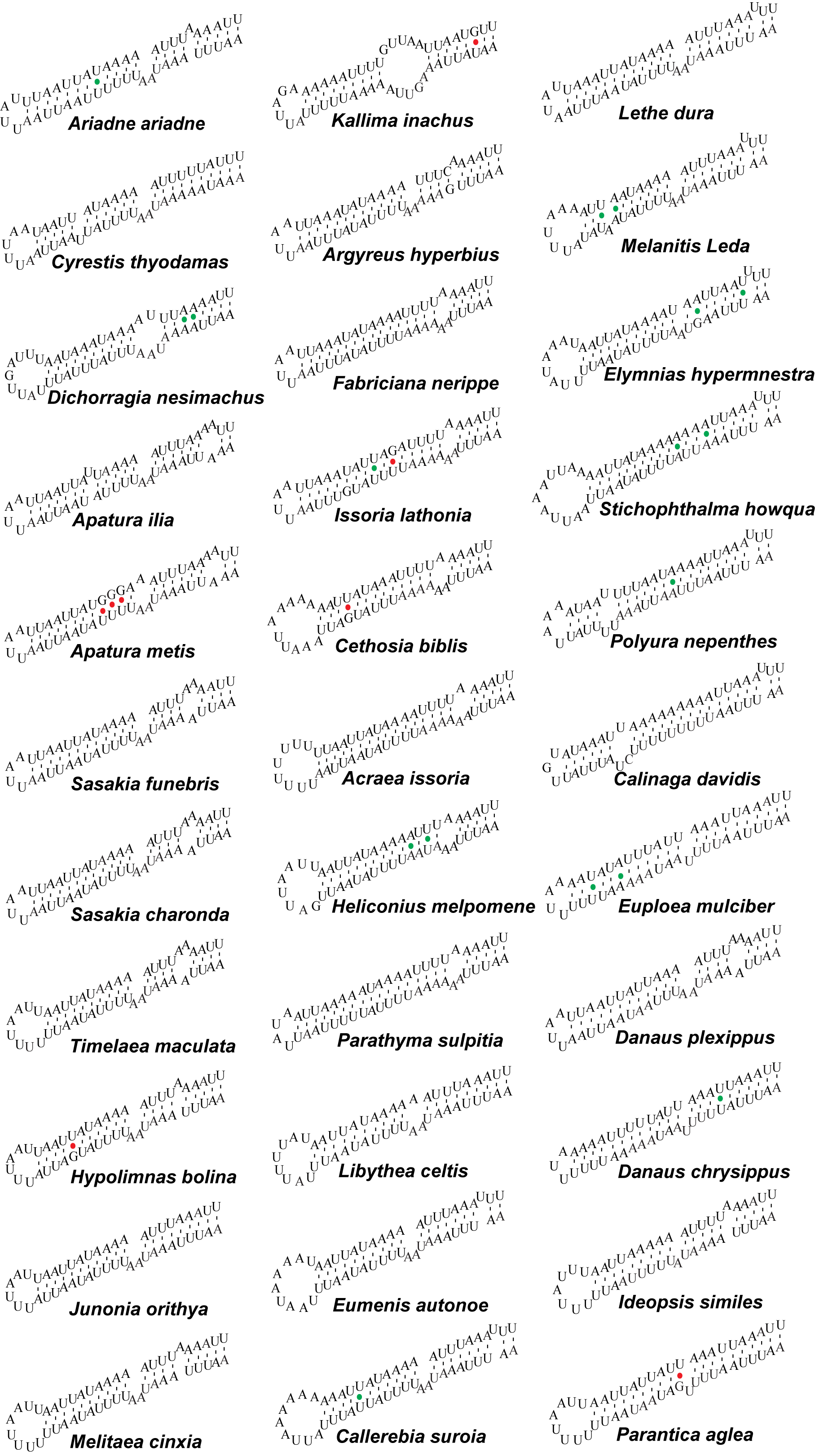

Supplement: S5 Fig — Symbols are as in S4 Fig. (TIF) [file pone.0124349.s005.tif]

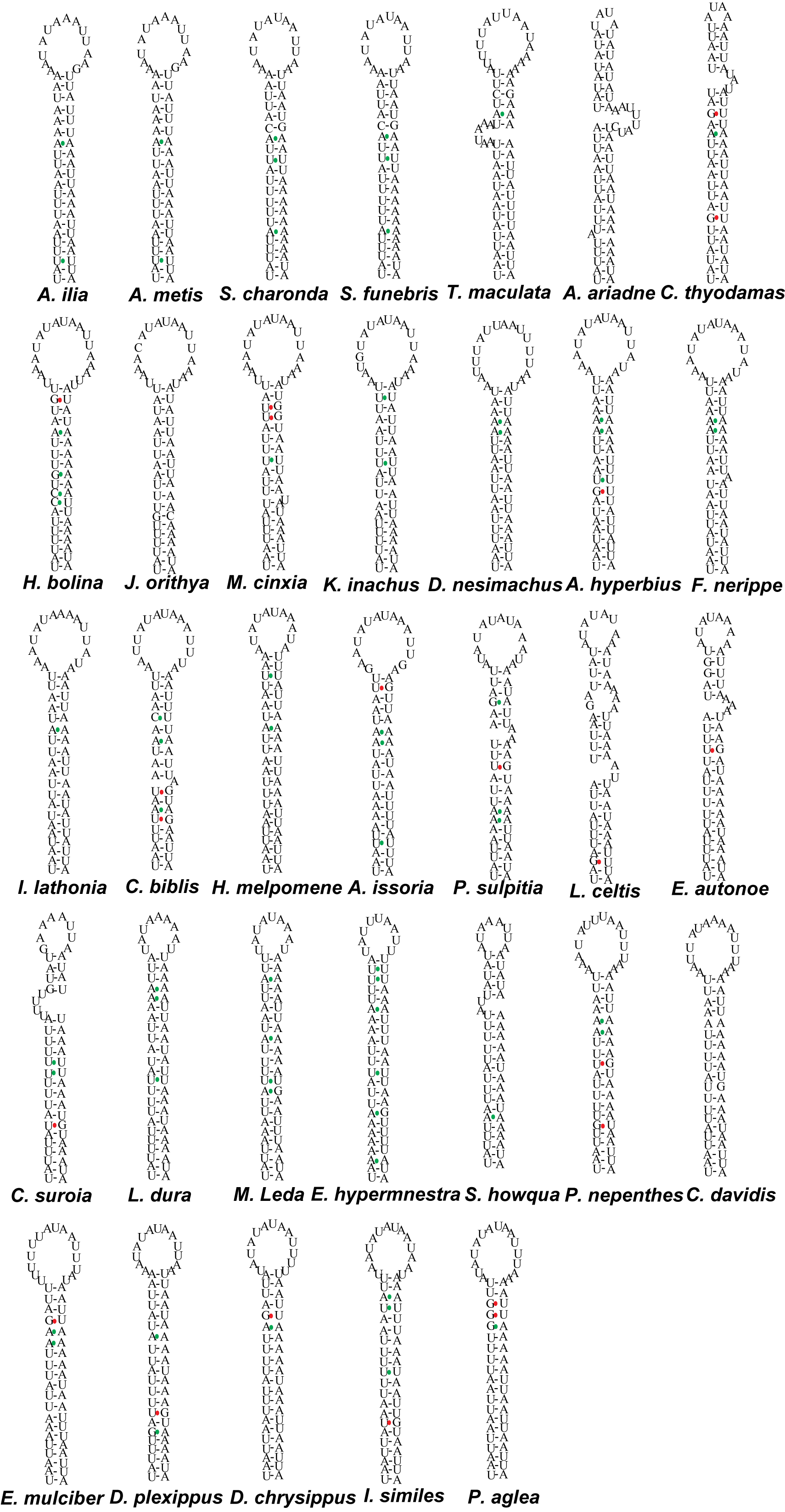

Supplement: S6 Fig — Symbols are as in S4 Fig. (TIF) [file pone.0124349.s006.tif]
