# Supplementary material for: Morphological Characters Are Compatible with Mitogenomic Data in Resolving the Phylogeny of Nymphalid Butterflies (Lepidoptera: Papilionoidea: Nymphalidae)
Source: PLoS One. 2015 Apr 10;10(4):e0124349. doi: 10.1371/journal.pone.0124349 (PMC4393276; doi:10.1371/journal.pone.0124349)
Supplement: S1 Table — (PDF) [file pone.0124349.s007.pdf]

Table S1. Materials and their sources in this study.

**Subfamily**	**Species**	**Sampling locality**	**Sampling time**
Charaxinae	*Polyura nepenthes*	Yandangshan, Zhejiang, China	July 2008
Heliconiinae	*Cethosia biblis*	Jiulianshan, Jiangxi, China	August 2009
Biblidinae	*Ariadne ariadne*	Jianfengling, Hainan,China	August 2008
Nymphalinae	*Hypolimnas bolina*	Jiulianshan, Jiangxi, China	August 2009
Pseudergolinae	*Dichorragia nesimachus*	Huangshan, Anhui, China	August 2010
Morphinae	*Stichophthalma howqua*	Dujiangyan, Sichuan, China	July 2006
Cyrestinae	*Cyrestis thyodamas*	Qiongzhong, Hainan, China	May 2009
Danainae	*Parantica aglea*	Qiongzhong, Hainan, China	May 2009
Satyrinae	*Elymnias hypermnestra*	Jianfengling, Hainan, China	August 2008
	Lethe dura	Qingchengshan, Sichuan, China	May 2012
	Callerebia suroia	Lijiang, Yunnan, China	July 2006
