# Supplementary material for: Morphological Characters Are Compatible with Mitogenomic Data in Resolving the Phylogeny of Nymphalid Butterflies (Lepidoptera: Papilionoidea: Nymphalidae)
Source: PLoS One. 2015 Apr 10;10(4):e0124349. doi: 10.1371/journal.pone.0124349 (PMC4393276; doi:10.1371/journal.pone.0124349)
Supplement: S2 Table — (PDF) [file pone.0124349.s008.pdf]

Table S2. Predicted initiation and termination codons distribution of the 13 mitochondrial PCGs among Nymphalidae species in this study.

Species	Start codon						Stop codon			
	ATA	ATT	ATC	ATG	CGA	TTG	TAA	TAG	TA	T
Apatura ilia	2	3	0	7	1	0	6	2	0	5
Apatura metis	1	4	0	7	1	0	9	1	0	3
Sasakia charonda	6	2	0	5	0	0	10	1	0	2
Sasakia funebris	3	3	0	6	1	0	8	1	0	4
Timelaea maculata	1	3	1	7	1	0	8	1	0	4
Ariadne ariadne	0	5	0	7	1	0	7	0	2	4
Cyrestis thyodamas	3	2	1	6	1	0	8	0	0	5
Kallima inachus	1	3	0	8	1	0	8	2	1	2
Melitaea cinxia	0	5	0	7	1	0	9	0	2	2
Hypolimnas bolina	0	4	1	7	1	0	7	1	1	4
Junonia orithya	0	4	1	7	1	0	9	0	2	2
Dichorragia nesimachus▼	3	1	0	7	1	0	10	1	0	2
Argyreus hyperbius	3	3	0	6	1	0	11	0	0	2
Acraea issoria	2	2	0	8	0	1	12	0	0	1
Fabriciana nerippe	1	5	0	6	0	1	8	0	2	3
Issoria lathonia	0	5	0	7	1	0	8	0	2	3
Heliconius melpomene	1	5	0	6	0	1	10	3	0	0
Cethosia biblis	1	4	1	6	1	0	6	1	3	3
Parathyma sulpitia	1	4	0	7	1	0	9	0	0	4
Libythea celtis	0	4	1	7	1	0	8	1	0	4
Eumenis autonoe	0	2	3	7	1	0	10	0	0	3
Melanitis leda	0	2	2	8	1	0	8	0	0	5
Elymnias hypermnestra	1	2	2	7	1	0	8	0	0	5
Lethe dura	2	4	0	6	1	0	7	0	0	6
Callerebia suroia	2	3	1	6	1	0	7	1	0	5
Stichophthalma howqua▼	1	3	1	6	1	0	11	0	1	1
Polyura nepenthes	1	4	1	6	1	0	10	0	1	2
Calinaga davidis	0	4	1	7	0	1	11	0	1	1
Euploea mulciber	1	4	0	7	1	0	8	1	0	4
Danaus plexippus	1	4	0	7	1	0	9	0	0	4
Danaus chrysippus	0	5	0	7	1	0	9	0	0	4
Ideopsis similes	2	4	0	6	1	0	8	1	0	4
Parantica aglea	2	2	2	6	1	0	7	2	0	4

▼ indicates lack of initiation codon for *nad2* gene in the mitogenome.
